# Wee1 kinase alters cyclin E/Cdk2 and promotes apoptosis during the early embryonic development of *Xenopus laevis*

**DOI:** 10.1186/1471-213X-7-119

**Published:** 2007-10-25

**Authors:** Brian N Wroble, Carla V Finkielstein, Jill C Sible

**Affiliations:** 1 Department of Biological Sciences, Virginia Polytechnic Institute and State University, Blacksburg, VA, USA

## Abstract

**Background:**

The cell cycles of the *Xenopus laevis *embryo undergo extensive remodeling beginning at the midblastula transition (MBT) of early development. Cell divisions 2–12 consist of rapid cleavages without gap phases or cell cycle checkpoints. Some remodeling events depend upon a critical nucleo-cytoplasmic ratio, whereas others rely on a maternal timer controlled by cyclin E/Cdk2 activity. One key event that occurs at the MBT is the degradation of maternal Wee1, a negative regulator of cyclin-dependent kinase (Cdk) activity.

**Results:**

In order to assess the effect of Wee1 on embryonic cell cycle remodeling, Wee1 mRNA was injected into one-cell stage embryos. Overexpression of Wee1 caused cell cycle delay and tyrosine phosphorylation of Cdks prior to the MBT. Furthermore, overexpression of Wee1 disrupted key developmental events that normally occur at the MBT such as the degradation of Cdc25A, cyclin E, and Wee1. Overexpression of Wee1 also resulted in post-MBT apoptosis, tyrosine phosphorylation of Cdks and persistence of cyclin E/Cdk2 activity. To determine whether Cdk2 was required specifically for the survival of the embryo, the cyclin E/Cdk2 inhibitor, Δ34-Xic1, was injected in embryos and also shown to induce apoptosis.

**Conclusion:**

Taken together, these data suggest that Wee1 triggers apoptosis through the disruption of the cyclin E/Cdk2 timer. In contrast to Wee1 and Δ34-Xic1, altering Cdks by expression of Chk1 and Chk2 kinases blocks rather than promotes apoptosis and causes premature degradation of Cdc25A. Collectively, these data implicate Cdc25A as a key player in the developmentally regulated program of apoptosis in *X. laevis *embryos.

## Background

The early *Xenopus laevis *embryo provides a rich context in which to investigate cell cycle regulation and the interplay between the cell cycle and development. The first twelve cleavage cycles following fertilization consist of rapid oscillations between S and M phase without intervening gap phases. These cell cycles do not engage checkpoints in response to damaged or unreplicated DNA [1-3]. Rather, embryonic cells that have incurred such assaults to the genome die by a maternally regulated program of apoptosis during gastrulation [2-4]. Beginning at the midblastula transition (MBT), cell cycles lengthen, acquiring gap phases and operable cell cycle checkpoints [5,6]. Furthermore, damaged or unreplicated DNA may trigger abnormal development but generally will not induce apoptosis [2,3]. Although the molecular players in cell cycle remodeling during the early development of *X. laevis *have been well characterized, little is known about the underlying controls that govern these events.

Early embryonic cell cycles are regulated by three cyclin-dependent kinase (Cdk) complexes. Cyclin A/Cdk1 and cyclin B/Cdk1 are the M-phase Cdks, and cyclin E/Cdk2 is the S-phase Cdk [7,8], although their functions may overlap [9]. The activity of the mitotic Cdk complexes are controlled by cyclin synthesis and degradation and by inhibitory phosphorylations on threonine 14 and tyrosine 15 by Wee1 and Myt 1 kinases [10,11]. Phosphorylation-mediated inhibition of Cdks is counteracted by members of the Cdc25 family of phosphatases [12-14]. In *X. laevis*, Wee1 kinase is present in pre-MBT embryos, but degraded after the MBT [15]. Prior to the MBT in *X. laevis *embryos, Wee1 and Myt1 act in opposition to Cdc25C, inhibiting Cdk1 [10,11].

At the MBT, the profile of kinases and phosphatases regulating Cdk activity is modified. Both Cdc25C and Myt 1 persist at relatively constant levels. In contrast, Cdc25A levels drop beginning at the MBT and maternally encoded Wee1 disappears at gastrulation when it is replaced by the more active zygotic kinase, Wee2 [16]. It is likely that this change in the ratio of kinase to phosphatase activity operating on the Cdks is an integral component of cell cycle remodeling that initiates at the MBT. In previous studies that support this hypothesis, overexpression of Cdc25A accelerated [12], whereas overexpression of Wee2 lengthened cleavage cycles [16].

In addition to its role in promoting S phase, cyclin E/Cdk2 also serves a developmental function in early *X. laevis *embryos. Oscillations in cyclin E/Cdk2 activity constitute a maternal developmental timer that regulates the timing of the events of the MBT [9]. One of these events is the degradation of maternal cyclin E itself [9,17,18]. Inhibition of Cdk2 by the specific Cdk inhibitor, Δ34Xic1, lengthens cleavage and delays the onset of the MBT and the degradation of cyclin E [9]. Although cyclin E levels are constant throughout pre-MBT development, cyclin E/Cdk2 activity oscillates twice per cell cycle, independently of protein synthesis and the nucleo-cytoplasmic ratio [9,17,19]. However, other inhibitors of the MBT such as α-amanitin (blocks zygotic transcription) and cycloheximide (blocks protein synthesis) do not affect the timing of cyclin E degradation [9,20], suggesting that the cyclin E/Cdk2 timer regulates the MBT but not vice versa. Overexpression of cyclin E in the early embryo disrupts nuclear divisions and triggers apoptosis after the MBT [21]. These effects are independent of Cdk activity, suggesting further complexity of the role of cyclin E during early development. A better understanding of how the cyclin E/Cdk2 developmental clock is regulated should give insight into the mechanisms that drive cell cycle remodeling at the MBT.

It has been proposed that oscillatory activity of cyclin E/Cdk2 is governed by changes in phosphorylation state [18]. However, the kinases and phosphatases that govern cyclin E/Cdk2 activity in *X. laevis *embryos are unknown. Overexpression of Chk1 in *X. laevis *embryos, which activates Wee1 and inhibits Cdc25A and Cdc25C, delays both the MBT and cyclin E degradation [22], suggesting that the cyclin E/Cdk2 timer is regulated by its phosphorylation state. In mammalian cells, Wee1 inhibits cyclin E/Cdk2 [23-25], but it was previously unknown whether Wee1 regulated cyclin E/Cdk2 activity *in X. laevis*.

Wee1 is the opposing kinase of Cdc25, is degraded after the MBT, and functions to inhibit both cyclin B/Cdk1 in metazoans and cyclin E/Cdk2 in mammals. Therefore, we disrupted the balance of Cdk phosphatase and kinase activity by overexpressing Wee1 in *X. laevis *embryos to more closely identify its role in cell cycle remodeling during the early embryonic development of *X. laevis*. Our results indicate that an imbalance of Cdk-inhibitory activity triggers apoptosis, most likely through the disruption of the cyclin E/Cdk2 timer, since direct inhibition of cyclin E/Cdk2 also induces apoptosis. These data suggest that proper coordination of cell cycle remodeling events at the MBT is required for embryonic survival. By comparing the developmental effects of Cdk inhibitors that trigger apoptosis to those that inhibit apoptosis, we identified Cdc25A as an important predictor of developmentally regulated apoptosis in *X. laevis *embryos.

## Results

### Overexpression of Wee1 lengthens pre-MBT cell cycles

**Figure 1:**
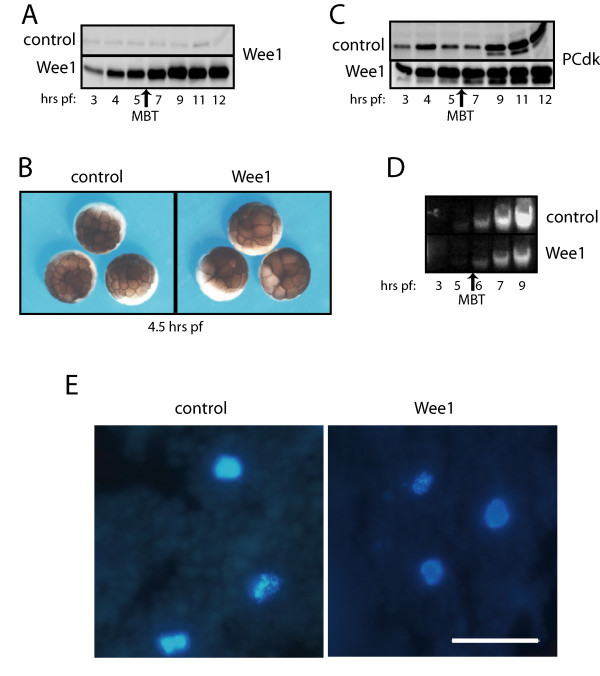
**Overexpression of Wee1 lengthens pre-MBT cell cycles**. One-cell stage embryos were microinjected with 2.5 ng mRNA encoding Wee1 or luciferase (control). (A) Embryos were collected at indicated times post-fertilization (hr pf) and analyzed for Wee1 protein by Western analysis. (B) *Xenopus *embryos injected with 2.5 ng Wee1 or luciferase mRNA were photographed at 4.5 hr pf, when embryos expressing exogenous Wee1 were delayed approximately one cell cycle compared to luciferase controls. (C) Overexpression of Wee1 results in premature tyrosine phosphorylation of Cdks. Embryo lysates were subjected to Western analysis using a phosphoCdk antibody (Cell Signaling Technology). Both phosphorylated p34Cdk1 and p32Cdk2, are recognized by this antibody, which likely accounts for the bands of slower and faster mobility, respectively. Phosphorylation of Cdks was apparent as early as Stage 6 (3 hrs pf) in embryos injected with Wee1. (D) Embryos (n = 10) were collected at the indicated stages, then DNA was isolated and resolved by agarose gel electrophoresis. Approximate timing of the MBT in luciferase expressing control embryos is indicated. E) Embryos were collected at Stage 9 (7 hrs pf), fixed, sectioned, stained with DAPI to visualize nuclear morphology, and photographed. A representative field is shown. scale bar = 50 μm

To determine the effect of disrupting the balance of Cdk kinase and phosphatase activities in embryos prior to the MBT, 2.5 ng mRNA encoding wild-type Wee1 or luciferase (control) was microinjected into one-cell stage embryos. Western analysis confirmed expression of exogenous Wee1 throughout the developmental stages examined (Figure 1A). Embryos expressing exogenous Wee1 exhibited slower cleavage cycles. At 4.5 hours post-fertilization (hrs pf), embryos expressing exogenous Wee1 were delayed by approximately one cell cycle compared to controls embryos injected with luciferase mRNA (Figure 1B) (Stage 6 = 32 blastomeres compared to Stage 7 = 64 blastomeres) [26]. Otherwise, these embryos developed with normal morphology through the MBT.

To determine whether the cell cycle lengthening induced by exogenous Wee1 coincided with the phosphorylation of Cdks, embryos expressing Wee1 or luciferase were assayed by Western analysis using a phosphoCdk primary antibody (Figure 1C). In untreated embryos, low-level tyrosine phosphorylation of Cdks occurs prior to the MBT then increases at the MBT concurrent with cell cycle lengthening [12,19,27]. Similarly, control embryos expressing luciferase demonstrated low levels of Cdk phosphorylation until the MBT. In contrast, Cdks were phosphorylated on tyrosine 15 as early as 3 hrs pf in embryos expressing exogenous Wee1, suggesting the cell cycle delay resulted from the inhibition of Cdks.

Mitotic cleavage cycles and nuclear cycles of DNA replication can be uncoupled in early *X. laevis *embryos [1]. Therefore, we wanted to determine whether exogenous Wee1 also affected DNA replication in early embryos. Total DNA was isolated from embryos expressing Wee1 or luciferase, resolved by gel electrophoresis and stained with ethidium bromide. Embryos expressing exogenous Wee1 contained less DNA throughout early development, compared to luciferase controls (Figure 1D). However, the DNA content increased through the early development of embryos overexpresssing Wee1 indicative of continuing DNA replication. The rate of increase in DNA content was consistent with the lengthened cleavage cycles, suggesting that nuclear and cytoplasmic cycles were coordinated. To examine nuclear morphology, embryos were collected at several stages, sectioned and stained with DAPI. Representative fields from embryos collected at Stage 9 are shown in Figure 1E. Embryos overexpressing Wee1 contained interphase and mitotic nuclei with normal morphology. There was no loss of nuclei as seen in embryos overexpressing cyclin E [21], although there were fewer and larger cells in these embryos, consistent with the delayed cell cycles. These results indicate that the overexpression of Wee1 delays both nuclear and cleavage cycles, but these cycles appear to remain coupled.

### Overexpression of Wee1 triggers apoptosis after the MBT

In previous studies, we showed that *X. laevis *embryos expressing exogenous checkpoint kinases, Chk1 or Chk2, exhibited a similar cell cycle delay and premature Cdk phosphorylation [28,29]. Furthermore, embryos expressing exogenous Chk2 developed normally through neurulation and beyond, and exogenous Chk2 protects embryos form apoptosis induced by ionizing radiation [29].

**Figure 2:**
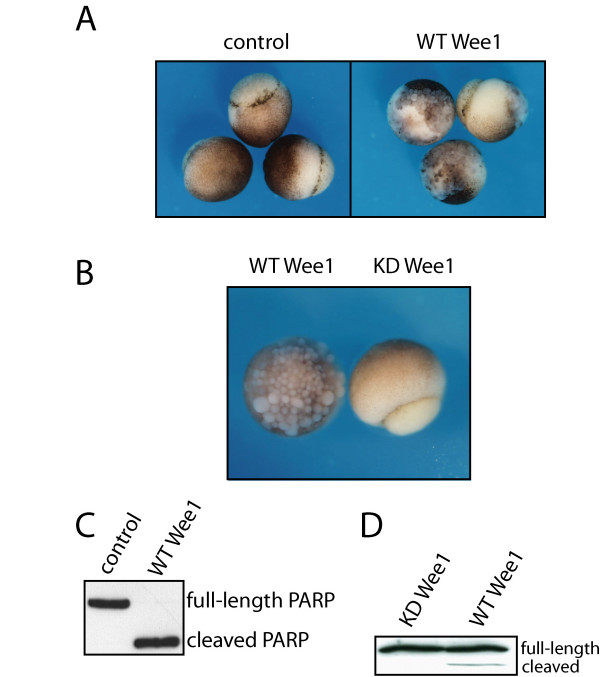
**Overexpression of Wee1 in early *Xenopus *embryos triggers apoptosis after the MBT**. One-cell stage *Xenopus *embryos were microinjected with 2.5 ng wild-type (WT) Wee1, kinase-dead (KD) Wee1, or luciferase (control) mRNA. (A-B) Embryos expressing Wee1, KDWee1, and luciferase developed normally until the early gastrula stage, when embryos expressing exogenous WT Wee1 exhibited abnormal morphology consistent with apoptosis. Embryos shown were photographed at gastrulation (Stage 11). (C-D) Embryo extracts were collected at Stage 11 and incubated with recombinant human PARP, a substrate for caspase 3 [4]. The presence of a cleaved PARP fragment indicates caspase activity, a marker of apoptosis.

Embryos overexpressing Wee1 developed normally through the MBT until early gastrulation (~Stage 10.5). However, during gastrulation, these embryos began to appear abnormal, exhibiting a loss of cellular attachment and overall organization (Figure 2A). The gross morphology of embryos expressing exogenous Wee1 appeared consistent with that of embryos that had undergone apoptosis in response to ionizing radiation [2,3], unreplicated DNA, or expression of dominant-negative Chk1/Chk2 [4,29].

To determine whether the kinase activity of Wee1 was required for its disruption of development, embryos were microinjected with wild-type (WT) or kinase-dead (KD) Wee1 mRNA (plasmids provided by Paul Mueller, University of Texas, San Antonio) [16]. The embryos expressing KD Wee1 developed normally through gastrulation (Figure 2B), indicating that kinase activity is required for the effects of exogenous Wee1.

To determine whether overexpression of Wee1 induced apoptosis, embryos were assayed for caspase activity in embryo lysates by the cleavage of exogenous human PARP protein [4]. Embryos expressing exogenous Wee1 were positive for caspase activity at gastrulation (Stage 11; ~12 hrs pf) indicated by the presence of a cleaved PARP fragment (Figure 2C). These results were surprising since exogenous Chk1 and Chk2, lengthen cleavage cycles and promote phosphorylation to the same extent as Wee1, yet exogenous Chk1 and Chk2 kinases inhibit rather than promote apoptosis [4,29]. Like the effects on gross morphology, the activation of caspases was dependent on Wee1 kinase activity as caspase activity was not detected in embryos expressing KD Wee (Figure 2D).

In *X. laevis *cell-free egg extracts, Wee1 accelerates apoptosis through interaction with an SH2 domain in the Crk-adaptor protein. Furthermore, Wee1 can restore apoptosis in extracts depleted of SH2 domain interactors [30]. To determine whether Wee1 associates with Crk in developing *X. laevis *embryos undergoing apoptosis, Wee1 was immunoprecipated from embryo extacts and the immunoprecipitates were immunoblotted for Crk. Constant levels of Crk were detected even in lysates overexpessing Wee1 (data not shown). This data indicate an association between Crk and Wee1 *in vivo*, and that all available Crk may be complexed with endogenous Wee1.

### Overexpression of Wee1 promotes the persistence of Cdc25A, cyclin E and Cdk2 activity

In *Xenopus *embryos, the MBT delineates a point when a host of cell cycle remodeling events occur. Among these remodeling events are the degradation of maternal Cdc25A [12] and cyclin E [19,31]. In previous studies, we showed that overexpression of Chk1/Chk2 causes premature degradation of Cdc25A and delayed degradation of cyclin E [22,29]. Furthermore, the transient activation of Chk1 at the MBT is required for the degradation of Cdc25A [32]. Since overexpression of Chk1, Chk2, and Wee1 all lengthen the cell cycle and trigger premature phosphorylation of Cdks, we wanted to investigate the effect of exogneous Wee1 on the timing of Cdc25A and cyclin E degradation, two hallmarks of the MBT.

**Figure 3:**
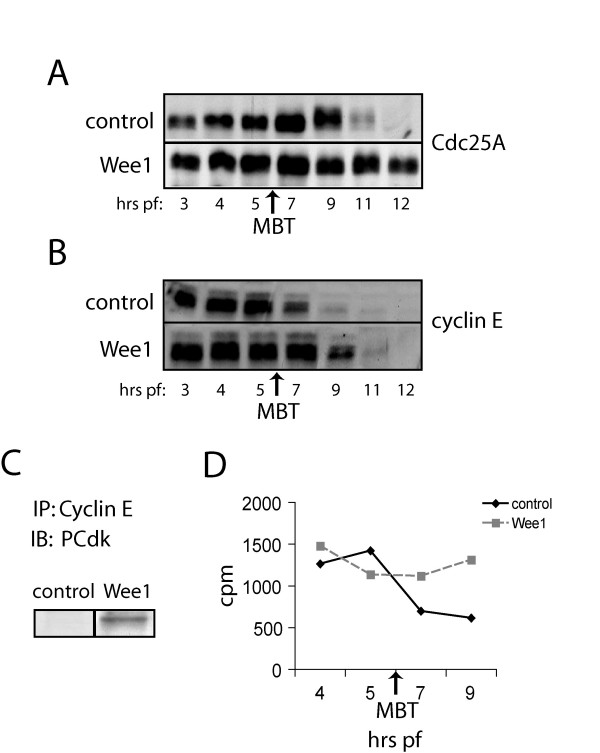
**Overexpression of Wee1 delays the MBT and alters Cdk2**. Embryos were injected at the one-cell stage with 2.5 ng of Wee1 or luciferase (control) mRNA. (A, B) Embryos were collected at the indicated stages and lysates were subjected to Western analysis using (A) Cdc25A and (B) cyclin E antibodies. Approximate timing of the MBT in luciferase expressing control embryos is indicated. (C) Embryos were collected at 4 hrs pf and lysates were immunoprecipitated with cyclin E serum. Immunoprecipitates were subjected to Western analysis using a primary phosphoCdk antibody (Cell Signaling Technology). (D) Cyclin E immunoprecipitates were collected at the times indicated and assayed for Cdk2 activity by phosphoryation of histone H1. The amount of γ-^32^P ATP incorporated in histone H1 is expressed as radioactive counts per minute (CPM).

Messenger RNA encoding wild-type Wee1 or luciferase (control) was microinjected into one-cell stage embryos, then embryos were collected at the indicated times and subjected to Western analysis of Cdc25A and cyclin E. Unlike Chk1/Chk2, overexpression of Wee1 resulted in delayed rather than accelerated degradation of Cdc25A (Figure 3A).

Additionally, overexpression of Wee1 delayed the degradation of cyclin E until mid-gastrulation (Figure 3B), similar to overexpression of Chk1 [22] and the expression of exogenous Δ34-Xic1 [9]. Both Chk1 and Δ34-Xic1 inhibit cyclin E/Cdk2 in embryos and are the only reagents known to delay the timing of cyclin E degradation in *X. laevis *embryos. This evidence and the prediction of our mathematical model suggest that timing of cyclin E degradation is determined by cyclin E/Cdk2 activity itself [18]. Therefore, we hypothesized that Wee1 may also inhibit cyclin E/Cdk2 *in X. laevis *embryos.

Inhibition of Cdk1 by Wee1 has been well characterized in *X. laevis *and other systems [10,33-35]. In human HeLa cells, Wee1 also phosphorylates Cdk2, inhibiting entry into S-phase [23-25]. In order to determine whether Wee1 inhibits Cdk2 in early *X. laevis *embryos, cyclin E was immunoprecipitated from embryos overexpressing Wee1, and Western analysis was performed on the immunoprecipitates to detect inhibitory tyrosine phosphorylation on Cdk2. Embryos overexpressing Wee1 expressed higher levels of phosphorylated Cdk2 (PCdk) indicating inhibition of Cdk2 by Wee1 (Figure 3C). These results suggest that the delays in degradation of cyclin E and Cdc25A by the overexpression of Wee1 could be due to the inhibition of Cdk2. The inhibition of cyclin E/Cdk2 is also consistent with the reduced DNA content in embryos expressing Wee1, since cyclin E/Cdk2 promotes the initiation of DNA replication during S phase [36,37].

To more directly measure Cdk activity, cyclin E immunoprecipitates were tested for kinase activity against histone H1. Immunoprecipatates were incubated with histone H1 and γ-^32^P ATP, reactions were resolved by polyacrylamide gel electrophoresis, H1 bands were excised, and counted by scintillation counting (Figure 3D). Despite the increased tyrosine phosphorylation of Cdk2 in embryos overexpressing Wee1, Cdk2 kinase activity was not repressed. In fact, Cdk2 activity persisted in these embryos after it had decreased after the MBT in control embryos. The persistence of Cdk2 is consistent with the delay in cyclin E degradation (Figure 3B). However, it was surprising that Cdk2 activity was not reduced since we had observed tyrosine phosphorylation of Cdk2 (Figure 3C) and this phosphorylation event is known to inhibit Cdk2 activity [28,36]. One explanation may be that exogenous Wee1 only phosphorylates a fraction of Wee1. Whether this fraction represents a specific pool that is inhibited or represents a steady state of total Cdk2 is not known.

### Inhibition of Cdk2 Triggers Apoptosis

Overexpression of Wee1 in *Xenopus *embryos resulted in a post-MBT apoptotic death. Since the overexpression of Wee1 not only alters cell cycle remodeling events (Figure 3) and the concentration of DNA at the MBT (6 hr pf; Figure 1D), but also phosphorylates Cdk2 (Figure 3), we wanted to determine whether inhibition of Cdk2 contributed to the induction of apoptosis. In order to inhibit Cdk2 directly, one-cell stage embryos were injected with Δ34-Xic1 protein or p27Xic1CK- protein as a control. The Δ34-Xic1 protein is a truncated form of the full length-Xic1 that specifically inhibits the activity of cyclin E/Cdk2 in *X. laevis *[38]. In *X. leavis *embryos, the expression of Δ34-Xic1 delays the degradation of cyclin E, thereby disrupting the cyclin E/Cdk2 timer [9]. The p27Xic1CK- construct contains four point mutations in the Cdk2 binding site that inhibit p27Xic1/Cdk2 interaction, thus serving as a negative control.

**Figure 4:**
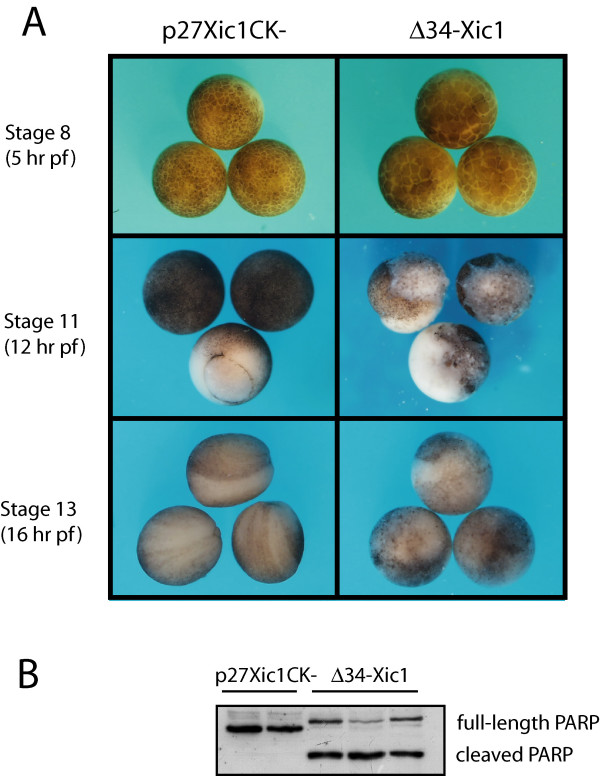
**Inhibition of Cdk2 triggers apoptosis**. One-cell stage embryos were injected with 5 ng of Δ34-Xic1 or p27Xic1CK- (control) protein. (A) Embryos expressing Δ34-Xic1 were delayed approximately one cell cycle at Stage 8 (5 hr pf) compared to p27Xic1CK- controls. At Stage 11 (12 hr pf), embryos expressing Δ34-Xic1 exhibited abnormal morphology consistent with apoptosis compared to p27Xic1CK- controls, which gastrulated. p27Xic1CK- controls continued to develop through gastrulation and formed a neural groove, evident at Stage 13 (16 hr pf). (B) Extracts of Stage 11 embryos were incubated with recombinant PARP, a substrate for caspase 3 [4]. The presence of a cleaved PARP fragment indicates caspase activity. Replicate samples are shown.

Embryos expressing Δ34-Xic1 were delayed slightly (~1 cell cycle at the MBT) compared to p27Xic1CK- controls (Figure 4A) but developed otherwise normally through the MBT. However, prior to gastrulation, embryos expressing Δ34-Xic1 died by apoptosis, indicated by gross morphology (Figure 4A) and activation of caspases (Figure 4B). In contrast, embryos injected with p27Xic1CK- protein persisted through gastrulation and neurulated (Figure 4A). These data indicate that specific inhibition of Cdk2 results in apoptosis in *Xenopus *embryos.

## Discussion

In this study, we demonstrate that altering the balance of Cdk tyrosine kinase and phosphatase activity in early *X. laevis *embryos leads to apoptosis after the MBT. We initiated these experiments because our previous studies suggested Chk1/Chk2 kinases function as inhibitors of apoptosis in early *X. laevis *embryos [4,29]. Expression of catalytically inactive, dominant-negative Chk1 or Chk2 in pre-MBT embryos induces apoptosis during early gastrulation [4,29]. Furthermore, wild-type Chk2 rescues embryos from radiation-induced apoptosis [29]. Because exogenous Chk2 lengthens cleavage cycles in a dose-dependent manner, one possible explanation for the protection from apoptosis was that longer cell cycles allowed time for repair of DNA damage. If this were the case, then Wee1, which also lengthens cleavage cycles due to tyrosine phosphorylation of Cdks, should also protect embryos from DNA damage-induced apoptosis.

However, the studies reported here yielded unexpected results and valuable information regarding the role and regulation of Cdk phosphorylation states in the early embryo. Wee1 mRNA lengthened cell cycles modestly, and until the MBT (Figure 1A), embryos overexpressing Wee1 were indistinguishable from those expressing low levels of exogenous Chk2 [29]. Both groups of embryos were delayed approximately one cell cycle when sibling controls reached the MBT. Furthermore, the extent of tyrosine phosphorylation was similar in embryos expressing exogenous Chk2 or Wee1. Despite these similarities, embryos injected with Wee1 mRNA died by apoptosis in the absence of any further perturbation.

Previous studies have implicated Wee1 as an inhibitor of Cdk2 activity in mammalian cells [23-25], but Wee1 had only been reported as a Cdk1 kinase in *X. laevis *embryos. In this study, we demonstrated that Wee1 delayed the degradation of cyclin E and phosphorylation of Cdk2, which functions as a developmental timer until the MBT. However, the catalytic activity of the total pool of cyclin E/Cdk2 was not reduced and persisted in embryos overexpressing Wee1, consistent with the cell cycle delay and delayed degradation of cyclin E. Therefore, like Chk1/Chk2, Wee1 causes tyrosine phosphoryation of Cdk1 and Cdk2 in *X. laevis *embryos. Nonetheless, despite similar effects on the cell cycle machinery, they have opposite effects regarding apoptosis in the embryo.

Because we discovered Wee1 to alter Cdk2 phosphorylation as well as trigger apoptosis, we tested whether apoptosis could be induced by a more specific Cdk2 inhibitor. A variant of Xic1 lacking the first 34 amino acids (Δ34-Xic1) is a specific stoichiometric inhibitor of cyclin E/Cdk2 [38] and delays both the degradaton of cyclin E and the onset of the MBT in *X. laevis *embryos [9]. In our studies, one hundred percent of embryos injected with exogenous Δ34-Xic1 underwent apoptosis after the MBT (Figure 4). Similarly, in breast cancer cells, transduction of a non-degradable form of the human homolog of Xic1, Kip1, induced cell cycle arrest, thereby inhibiting cellular proliferation. Furthermore, exogenous Kip1 caused an increase in the number of apoptotic cells [39].

## Conclusion

**Figure 5:**
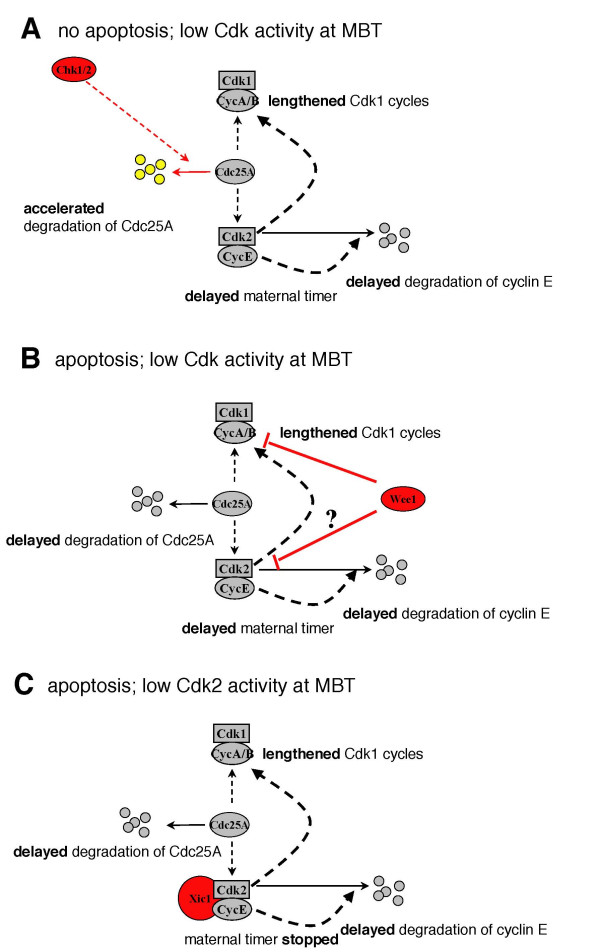
**Effects of inhibitors of Cdk activity on early development of *X. laevis***. (A) Exogenous Chk1/2 indirectly inhibits Cdk1 and Cdk2 by targeting Cdc25A for degradation. Onset of the MBT is delayed, but development proceeds without apoptosis. (B) Exogenous Wee1 phosphorylates Cdk1 and Cdk2. The total pool of Cdk1 activity is inhibited. Onset of the MBT including degradation of Cdc25A is delayed. Embryos die by apoptosis during early gastrulation. (C) Exogenous Δ34-Xic1 inhibits Cdk2 but not Cdk1. Like Wee1, Δ34-Xic1 delays the MBT including degradation of Cdc25A, and induces apoptosis.

Table 1 and Figure 5 summarize the effects of three different types of Cdk inhibitors on early development in *X. laevis *[4,29]. Exogenous Chk1/2, Wee1, and Δ34-Xic1 all modestly lengthen cleavage cycles, and delay the degradation of cyclin E, thus delaying the timing of the MBT. Whereas Chk1/2 and Wee1 inhibit Cdk1 and cause phosphorylation of Cdk2, Δ34-Xic1 is a specific inhibitor of Cdk2. These data stress the importance of cyclin E/Cdk2 in the timing of early developmental events.

**Table 1:** Effects of three Cdk inhibitors on early development of the *X. laevis *embryo

*effect on:*	*Chk1/2*	*Wee1*	*Δ34Xic1*
cleavage cycles	dose-dependent lengthening [28, 29]	modest lengthening	modest lengthening [9]
Cdk1 activity	inhibits via Tyr phosphorylation [28]	inhibits via Tyr phosphorylation [10]	no effect [38]
Cdk2 activity	inhibits via Tyr phosphorylation [29]	persists with Tyr phosphorylation	stoichiometric inhibitor [38]
degradation of cyclin E	delayed [28]	delayed	delayed [9]
degradation of Cdc25A	premature [22, 29, 32]	delayed	delayed
Cdk kinase:ppase activity at the MBT	increased kinase: decreased ppase [32, 40]	increased kinase: decreased ppase	unknown kinase: decreased ppase
effect on apoptosis	inhibits [4, 29]	activates	activates

Despite similar effects on development prior to the MBT, only Wee1 and Δ34-Xic1 induce apoptosis, whereas Chk1/Chk2 function as inhibitors of apoptosis. One possible explanation for this difference apparent in Table 1 is the effect of these reagents on Cdc25A levels at the MBT and consequently on the ratio of Cdk kinases:phosphatases at the MBT. Chk1/Chk2 trigger the premature degradation of Cdc25A before the MBT whereas Wee1 and Δ34-Xic1 delay the degradation of Cdc25A. These data suggest that Cdc25A may promote apoptosis and/or that a high Cdk kinase:phosphatase ratio inhibits apoptosis. In support of the former, exogenous non-degradable Cdc25A triggers cell death in the embryo during early gastrulation [32]. In support of the latter Chk1/Chk2 may activate Wee1 in *X. laevis*, leading to an even higher Cdk kinase:phosphatase level than would be achieved by the degradation of Cdc25A alone [40]. Alternatively, Chk1/Chk2 may block apoptosis by another pathway altogether, distinct from their effect on the cell cycle machinery. These studies illustrate that cell cycle remodeling events must be appropriately coordinated for the embryo to develop beyond the MBT. These studies also illustrate that cell cycle regulators that have been well characterized biochemically *in vitro *or in cell culture systems may have additional functions that can be uncovered in the rich context of the developing embryo.

## Methods

### Manipulation and maintenance of embryos

Eggs from wild-type *Xenopus laevis *(*Xenopus *Express) were fertilized *in vitro*, dejellied in 2% cysteine in 0.1× MMR (0.5 mM HEPES, pH 7.8, 10 mM NaCl, 0.2 mM KCl, 0.1 mM MgSO_4_, 0.2 mM CaCl_2_, 0.01 mM EDTA), and maintained in 0.1× MMR. Embryos were staged [26] and subjected to manipulation. Embryos were injected at the one-cell stage with specific concentrations of Wee1 or luciferase mRNA dissolved in 25–30 nL TE buffer (10 mM Tris, pH 8.0, 1 mM EDTA). In other experiments, embryos were injected with Δ34-Xic1 [38] and p27Xic1CK- protein diluted in buffer (20 mM Hepes, pH 7.5, 88 mM NaCl, 7.5 mM MgCl_2_, 10 mM β-mercaptoethanol). Δ34-Xic1 lacks the first 34 amino acids of the p27Xic1 protein. p27Xic1CK- has the conserved residue in the kinase domain mutated (R33A, L35A, F65A, F67A). Embryos were observed with an Olympus SZX12 stereomicroscope and photographed with an Olympus DP10 digital camera.

### *In Vitro *Transcription of Wee1 mRNA

Wee1 cDNA in pBluescript was kindly provided by Dr. Monica Murakami (National Cancer Institutes. Plasmids encoding Wee1 were linearized and used as templates to produce polyadenylated mRNA using the Ambion T3 mMessage mMachine *in vitro *transcription kit (Ambion). The control mRNA encoding exogenous luciferase protein was prepared as described previously [28]. Experiments with kinase-dead and wild-type Wee1 mRNA were performed with constructs provided by Dr. Paul Mueller (University of Texas at San Antonio). In the kinase-dead mutant, the codon for lysine 239 was converted to the codon for arginine.

### Western Analysis

Embryos were lysed in EB buffer (20 mM HEPES, pH 7.5, 80 mM β-glycerophosphate, 15 mM MgCl_2_, 20 mM EGTA, 50 mM NaF, 1 mM sodium orthovanadate, 1 mM dithiothreitol, 1 mM phenylmethylsulfonyl fluoride, 20 mg/mL leupeptin, 1 mM microcystin). Samples were then resolved on SDS polyacrylamide gels), transferred to a nitrocellulose membrane and blocked in 3% nonfat dry milk in TBS- 0.1%Tween (tris-buffered saline- 20 mM Tris-Base, 0.14 M NaCl, pH-7.6) or 5% BSA in TBS – 0.1% Tween. Membranes were incubated in primary antibody against Cdc25A and cyclin E (gifts from Dr. James Maller), and Wee1 (1:1000) (Zymed), diluted in 5% BSA-TBS- 0.1% Tween overnight at 4°C. Membranes were washed then incubated in secondary antibody (Peroxidase-conjugated AffiniPure Donkey Anti-Rabbit IgG (Jackson Immuno Research Laboratories Inc.) 1:10,000 in TBS- 0.1% Tween. Immunoreactive proteins were detected by chemilluminescence using an ECL Plus kit (Amersham).

### Isolation and visualization of genomic DNA

Embryos were injected with Wee1 or luciferase mRNA, collected at indicated times, and lysed in DNA digestion buffer (10 mM Tris, 100 mM EDTA, 50 μg/mL RNase, 0.5% SDS). Lysates were incubated at 37°C for 2 hrs. Proteinase K was then added to a final concentration of 100 μg/mL with continued incubation at 50°C for 4 hrs with slight intermittent manual agitation. Genomic DNA was phenol/chloroform extracted, and precipitated overnight at -20°C in 100% ethanol. Samples were resuspended in TE, and resolved on a 0.7% agarose gel, and ethidium bromide-stained DNA was visualized and photographed under UV illumination.

### Assessment of nuclear morphology

Embryos were collected at the times indicated, fixed in 4% paraformaldehyde, dehydrated through an ethanol series, cleared in CitriSolv (Fisher), embedded in paraffin, sectioned 7 μm thick, deparaffinized, rehydrated, and stained with 1 μg/ml DAPI. Serial sections were viewed and photographed on an Olympus AX70 fluorescence microscope equipped with a Color View 12 digital camera.

### Assay for cleavage of PARP

Embryos previously injected with Wee1 and luciferase mRNA or Δ34-Xic1 and p27Xic1CK- proteins were collected at desired stages (post-gastrulation), snap frozen on dry ice, and assayed for the cleavage of exogenous PARP [4]. Specifically, embryos were homogenized in caspase extraction buffer (10 μl/embryo) (CEB: 80 mM β-glycerophosphate, 15 mM MgCl_2_, 20 mM EGTA, 10 mM DTT). Lysates were incubated with 2 ng/mL recombinant human PARP (Alexis Biochemicals) at 27°C for 15 min, resolved on an SDS-polyacrylamide gel, and transferred to a nitrocellulose membrane. Anti-PARP (1:5000) and HRP-conjugated anti-rabbit (Cell Signaling Technology) (1:2000) diluted in 10% nonfat dry milk in PBS were used as primary and secondary antibodies, respectively. Both full-length and cleaved PARP proteins were detected by chemiluminescence using an ECL Plus kit (Amersham).

### Immunoprecipitation-Western analysis and kinase assays

Embryos were injected with Wee1 or luciferase mRNA, collected at indicated times, and lysed in EB. Antiserum against cyclin E (gift from Dr. James Maller) was used to immunoprecipitate cyclin E/Cdk2 or antibodies against Wee1 (Zymed) were used to precipitate Wee1 and associated proteins. Embryos lysates were precleared with protein G Sepharose beads (Sigma, St. Louis, MO) for 30 min, then mixed with antibodies, and incubated overnight on ice. Protein G Sepharose beads were added the next day and incubated with the immunoprecipitates for 1 h with rotation. Beads were then washed twice in low-salt buffer (20 mM Tris, pH 7.4, 5 mM EDTA, 0.1% Triton ×-100, 100 mM NaCl) and twice in high-salt buffer (20 mM Tris, pH 7.4, 5 mM EDTA, 0.1% Triton ×-100, 1 M NaCl). Immunoprecipitates were then mixed with 2× gel loading buffer (1× = 0.6 mM Tris base, 2% glycerol, 3% SDS, 0.002% bromphenol blue) containing 10 mM *n*-ethylmaleimide, and resolved on an SDS-polyacrylamide gel. Western analysis of cyclin E precipitates was performed as described using a primary phospho-Cdk antibody (Cell Signaling Technology) diluted 1:1000 or a monoclonal Crk antibody (BD Biosciences) diluted 1: 5000 in 5% BSA-TBS-Tween and a secondary HRP anti-rabbit or anti-mouse antibody (Cell Signaling Technology) diluted 1:2000 in 5% BSA-TBS- 0.1% Tween.

For kinase assays, immunoprecipitates were washed twice in kinase buffer (20 mM HEPES, pH 7.5, 15 mM MgCl2, 5 mM EGTA, 1 mM dithiothreitol), and then incubated 20 min at 27°C with 25 μl of kinase buffer containing 0.2 mg/ml bovine serum albumin, 0.5 mg/ml histone H1, 200 μM [γ-^32^P]ATP [2 cpm/fmol]). Reactions were terminated with 25 μl of 2× gel loading buffer containing 25% β-mercaptoethanol, heated at 95°C for 2 min, and resolved by PAGE. The gels were stained with Coomassie Blue and dried. Incorporation of [γ-^32^P]ATP was determined by Cerenkov scintillation counting of the excised histone H1 band.

### Generation and purification of p27Xic1CK- protein

Rosetta cells (Novagen) were transformed with pGEX vector encoding the GST-p27Xic1CK- containing 4 mutations in p27Xic1 (R33A, L35A, F65A, F67A). Bacterial pellets were resuspended in buffer × (50 mM Tris pH 8, 250 mM NaCl, 1 mM EDTA, 1 mM EGTA) containing 1 tablet of Complete protease inhibitors (Roche) and 1 mM DTT. Cell suspension was incubated at room temperature with 100 μg/mL lysozyme for 20 min with stirring then for an additional five minutes with 1/20 volume 10% Triton ×-100. Cell suspension was then sonicated 2 × 30 seconds and centrifuged at 17,510 × g for 1 hr at 4°C. The supernatant fraction was loaded onto a column of glutathione-agarose beads (Sigma) previously equilibrated with 5 volumes of extraction buffer (50 mM Tris pH 8, 250 mM NaCl, 1 mM EDTA, 1 mM EGTA). Supernatant was passed through the column twice. The column was then washed with 10 volumes of buffer A (50 mM Tris pH 8.0, 0.5 M NaCl, 1 mM EDTA, 1 mM EGTA, 5 mM DTT) and 10 volumes of buffer B (20 mM Tris pH 8.0, 1 M NaCl, 1 mM EDTA, 1 mM EGTA, 5 mM DTT). p27Xic1CK- protein was eluted with 5 mL buffer × containing 100 mM glutathione (GSH;Sigma), pH 8.0. Purified p27Xic1CK- was then concentrated by buffer exchange using Amicon Ultra Ultracel low binding regenerated cellulose tubes (Millipore, Bedford, MA), resuspended in buffer (20 mM Hepes pH 7.5, 88 mM NaCl, 7.5 mM MgCl_2_, 10 mM β-mercaptoethanol) and stored at -80°C.

## Abbreviations

Cdk; cyclin-dependent kinase

hr; hour

MBT; midblastula transition

pf; post-fertilization

## Competing interests

The author(s) declare that there are no competing interests.

## Authors' contributions

BNW designed and carried out the experiments and drafted the manuscript. CVF helped design and perform the Xic experiments and helped draft the manuscript. JCS participated in the project and experimental design and data interpretation, performed the nuclear morphology and kinase assays and drafted the manuscript. All authors have read and approved the final version of the manuscript.
